# Dihydrotanshinone Inhibits Hepatocellular Carcinoma by Suppressing the JAK2/STAT3 Pathway

**DOI:** 10.3389/fphar.2021.654986

**Published:** 2021-04-29

**Authors:** Xue Hu, Fangzhou Jiao, Lan Zhang, Yingan Jiang

**Affiliations:** ^1^ Department of Infectious Diseases, Renmin Hospital of Wuhan University, Wuhan, China; ^2^ Department of Gastroenterology, Renmin Hospital of Wuhan University, Wuhan, China

**Keywords:** dihydrotanshinone, hepatocellular carcinoma, JAK2, stat3, apoptosis

## Abstract

Liver cancer is the sixth most commonly diagnosed cancer and the fourth leading cause of cancer death. Most (75–85%) primary liver cancers occurring worldwide are hepatocellular carcinoma (HCC). The development of resistance and other drug related side effects are the prime reasons for the failure of treatment. Therefore, developing high-efficacy and low-toxicity natural anticancer agents is greatly needed in the treatment of HCC. Dihydrotanshinone (DHTS) is widely used for promoting blood circulation and antitumor. The aim of the present study was to investigate the effect and mechanism of DHTS-induced apoptosis of HCC, both *in vitro* and *in vivo*. We found that DHTS inhibited the growth of several HCC cells (HCCLM3, SMMC7721, Hep3B and HepG2). DHTS induced the apoptosis of SMMC7721 cells. Immunofluorescence results have showed that DHTS decreased STAT3 nuclear translocation. Moreover, Western blot results have demonstrated that DHTS suppressed the activation of JAK2/STAT3 signaling pathway. In addition, xenograft results have showed that DHTS suppressed tumor growth of SMMC7721 cells *in vivo* by inhibiting the p-STAT3. Thus, we demonstrated that DHTS could inhibit HCC by suppressing the JAK2/STAT3 pathway. DHTS has potential to be a chemotherapeutic agent in HCC and merits further clinical investigation.

## Introduction

Liver cancer is the sixth most commonly diagnosed cancer and the fourth leading cause of cancer death (Huang et al., 2020). Most (75–85%) primary liver cancers occurring worldwide are hepatocellular carcinoma (HCC) (Huang et al., 2020). HCC is one of the most common malignancies worldwide, particularly in many Asian and Eastern Africa (Kutluet et al., 2018; Yang et al., 2019; Huang et al., 2020). The treatment options for HCC mainly include surgical resection, transarterial chemoembolization, ethanol or acetic acid injection and, in rare cases, liver transplantation (Yu et al., 2016). Although surgical resection is a recommended treatment opinion, most patients are not eligible for surgical resection because of poor hepatic condition or tumor extent (Tabrizian et al., 2015; Yu et al., 2016). And 40–70% of patients relapse within 5 years of removal of HCC (Wen et al., 2018). Systemic chemotherapy is another possible common therapy. However, the development of resistance and other drug related side effects are the prime reasons for the failure of treatment (Cao et al., 2017). Therefore, developing high-efficacy and low-toxicity natural anticancer agents is greatly needed in the treatment of HCC.

Dihydrotanshinone (DHTS) is the main components isolated from *Salvia miltiorrhiza Bunge*, a functional food in Asian countries (Wei et al., 2016). It has been demonstrated that DHTS is widely used for promoting blood circulation and antitumor (Wang et al., 2015; Tan et al., 2020). Recent studies have reported that DHTS could inhibit cells proliferation or induce apoptosis in various tumor cells (Liu et al., 2015; Chen et al., 2017; Cai et al., 2018; Wang et al., 2020). However, the molecular mechanisms underlying DHTS-induced apoptosis in HCC still needs further investigation.

The Janus-activated kinase (JAK) proteins are a family of cytosolic tyrosine kinases (Honda et al., 2017). JAK2, one of a family of four JAK proteins, directly phosphorylate STAT3 at Y705 (Hu et al., 2018). Signal transducers and activators of transcription (STAT) proteins compose a family of transcription factors (Cafferkey and Chau, 2016). STAT3 is a prominent member in this family and a main survival signaling pathway in malignancy (Zhu et al., 2016). Furthermore, JAK2/STAT3 signaling pathway involved in regulating the proliferation and apoptosis in a variety of cancer cells (Jiang et al., 2016; Iriki et al., 2017; Wu et al., 2017).

Till date, few researchers focus on the relationship between DHTS and HCC, and the molecular mechanisms underlying any interaction have not been established. Therefore, this paper reports that DHTS is involved in inhibiting the growth of HCC cells via suppressing the JAK2/STAT3 pathway.

## Materials and Methods

### Cell Culture and Treatments

Human HCCLM3, SMMC7721, Hep3B and HepG2 cells were purchased from the Type Culture Collection of the Chinese Academy of Sciences. HCCLM3, Hep3B and HepG2 cells were grown in DMEM (Gibco, United States) containing 10% heat-inactivated fetal bovine serum (FBS) (GIBCO, United States) and 1% penicillin/streptomycin (Sigma-Aldrich, United States). SMMC7721 were grown in 1640 (Gibco, USA) with 10% FBS and penicillin/streptomycin. The cells were maintained at 37°C in a humidified incubator containing 95% air and 5% CO2.

### Reagents and Antibodies

Dihydrotanshinone (DHTS) was purchased from Sigma-Aldrich (USA). DHTS was dissolved in DMSO to form a concentration of 10 μM stock solution. Anti- STAT3 (#9139), Phospho- Stat3 (Tyr705) (#9145), JAK2 (#3230), Phospho-Jak2 (Tyr1007/1008) (#3771), Bax (#5023), Bcl-2 (#), Cyt C (#4280), Survivin (#2808), cleaved caspase-3 (#9661), cleaved caspase-9 (#20750), cleaved caspase-7 (#8438) were from Cell Signaling Technology (United States). The secondary antibodies were purchased from LI-COR (United States).

### Cell Viability Assay *in Vitro*

The viability and inhibition of Cells was measured by the Cell Counting kit- 8 assay (CCK- 8 assay, Dojindo, Japan). Cells (5 × 10^3^ cells) were seeded into a 96- well microtiter plate per well. Then, HCCLM3, SMMC7721, Hep3B cells were treated with DHTS (1, 2, 4, 8, 16, or 32 μg/ml) for 24 h HepG2 cells were treated with DHTS (5, 10, 20, 40, 80, 160 μg/ml) for 24 h. Subsequently, 10 μL CCK-8 dye was added to each well and incubated for 2 h at 37°C. Then The absorbance was measured at a wavelength of 450 nm using an iMark microplate reader (Victor3 1420 Multilabel Counter, PerkinElmer).

### Hoechst 33258 Staining

Hoechst 33258 Assay was purchased from Beyotime Institute of Biotechnology (Shanghai, China). Hoechst 33258 is a blue fluorescent dye which is used for the detection of cells apoptosis. SMMC7721 were seeded into a sterile 6-well plate for 24 h. Subsequently, DHTS (1, 2, 4 μg/ml) was added for another 24 h. Then, the cells were fixed in 4% paraformaldehyde solution for 30 min and incubated with Hoechst 33258 dye for 10 min. After washing three times with PBS, apoptotic morphological features were examined and photographed under a fluorescence microscope (OLYMPUS, Japan).

### Flow Cytometric Analysis for Apoptosis

Cells were cultured in 6-well plates for 24 h, Then, the cells were treated with DHTS (1, 2, 4 μg/ml) for 24 h. After treatment, cells were collected and washed twice with cold PBS. Then, cells were resuspended in binding buffer at a concentration of 1 × 10^6^ cells/ml. Next, 5 μL Annexin V-PE and 10 μL 7-AAD or 50 μL PI staining (for cell cycle detection) were added in the cell suspension and incubated in the dark for 15 min at room temperature. The percentage of apoptotic cells was evaluated by flow cytometric analysis (FACSCalibur, Becton-Dickinson).

### Western Blot Analysis

The harvested cells were lyzed with cold RIPA buffer (Beyotime, China) containing protease and phosphatase inhibitors and PMSF (Beyotime, China) and total proteins were extracted. The protein samples (30 µg) were separated using 12% SDS-PAGE and transferred onto polyvinylidene difluoride (PVDF) membrane (Millipore, Unites States). Membranes were blocking with 5% milk at room temperature for 1 h. After washing three times with PBS, the bands were incubated at 4°C overnight with the different primary antibodies at the recommended concentrations. After three washes with PBST, the bands were incubated with the secondary fluorescent antibody at 37°C in the dark for 1 h. After washing three times, the bands were analyzed using the Odyssey Infrared Imaging system (LI-COR, Unites States).

### Immunofluorescence

After treatment, cells were fixed with 4% paraformaldehyde for 30 min at room temperature. Cells were then permeabilized with 0.2% Triton X-100 for 15 min. After three washes, the permeabilized cells were blocked with 5% bovine serum albumin (BSA) for 30 min. Then, cells were incubated with 1:100 dilution of Stat3 (mouse antibody) overnight at 4°C. The next day, cells were incubated in a 1:100 dilution of goat anti-mouse Ig G antibody (Invitrogen) for 1 h at room temperature. The nuclei were counterstained using DAPI (Beyotime, China). Images of cells were visualized under fluorescence microscope (Olympus, Japan).

### Lentiviral Transduction and Generation of Stable Cell Lines.

The STAT3 plasmid, STAT3 shRNA plasmid, and control plasmid were purchased from Miaoling Bioscience and Technology (Wuhan, China). All plasmids contain enhanced green fluorescent protein (EGFP) and anti-puromycin gene site. The STAT3 plasmid, STAT3 shRNA plasmid, or NC plasmid was co-transfected with packaging plasmids pSPAX2 (#12260, addgene) and pMD2. G (#12259, addgene) into HEK293T cells by using transfection reagent Lipofectamine 2000 (Invitrogen) to produce lentiviruses. After transfection, the supernatants containing the lentiviruses were collected and used to infect SMMC7721. The expression of EGFP of cells was used to evaluate the efficiency of viral infection by using fluorescence microscope. 5 mg/ml puromycin was added into medium to select the successfully transduced cells. Western blotting was performed to evaluate efficiency of transfection.

### Xenograft Tumor Model

Male BALB/c nude mice, 5–6 weeks of age, were purchased from Beijing HFK Experimental Animal Center (Beijing, China). All procedures of the mice were approved by the Committee on the Ethics of Animal Experiments and performed in accordance with National Institutes of Health (NIH) Guide for the Care and Use of Laboratory Animals. All mice were kept in specific pathogen-free (SPF) animal house and fed with autoclaved food, sterile water, and filtered air. Nude mice were randomly divided into four groups, and each group contained 6 mice. Mice were subcutaneously inoculated SMMC7721 cells on the lower right flank. The xenograft tumors were allowed to grow until 100 mm^3^ in size before giving the treatment of DHTS (5, 10, or 15 mg/kg, via intraperitoneal injection) thrice a week. The tumor volume (TV) was calculated according to the following formula: TV (mm3) = d2 × D/2 (d and D are the shortest and longest diameters, respectively). TV was measured every alternate day.

### TUNEL Staining

The cell death of the xenograft tumors was detected by TUNEL kits (Roche, United States). Briefly, harvested tumors were fixed in 4% paraformaldehyde, and sliced into 4 μm thickness. Subsequently, the sections were deparaffinized and rehydrated. Then, the sections were incubated with TUNEL according to the manufacturer’s instructions. The TUNEL-positive cells were observed and analyzed under a fluorescence microscope (Olympus, Japan).

### Statistical Analysis

Experimental results were represented as mean ± standard deviation and analyzed using the IBM Statistical Product and Service Solutions (SPSS) software. Statistical differences of the results between groups were performed with one-way ANOVA test and Student’s test. Results were considered statistically when *p* value was less than 0.05.

## Results

### Dihydrotanshinone Inhibits the Proliferation of Hepatocellular Carcinoma Cells

**FIGURE 1:**
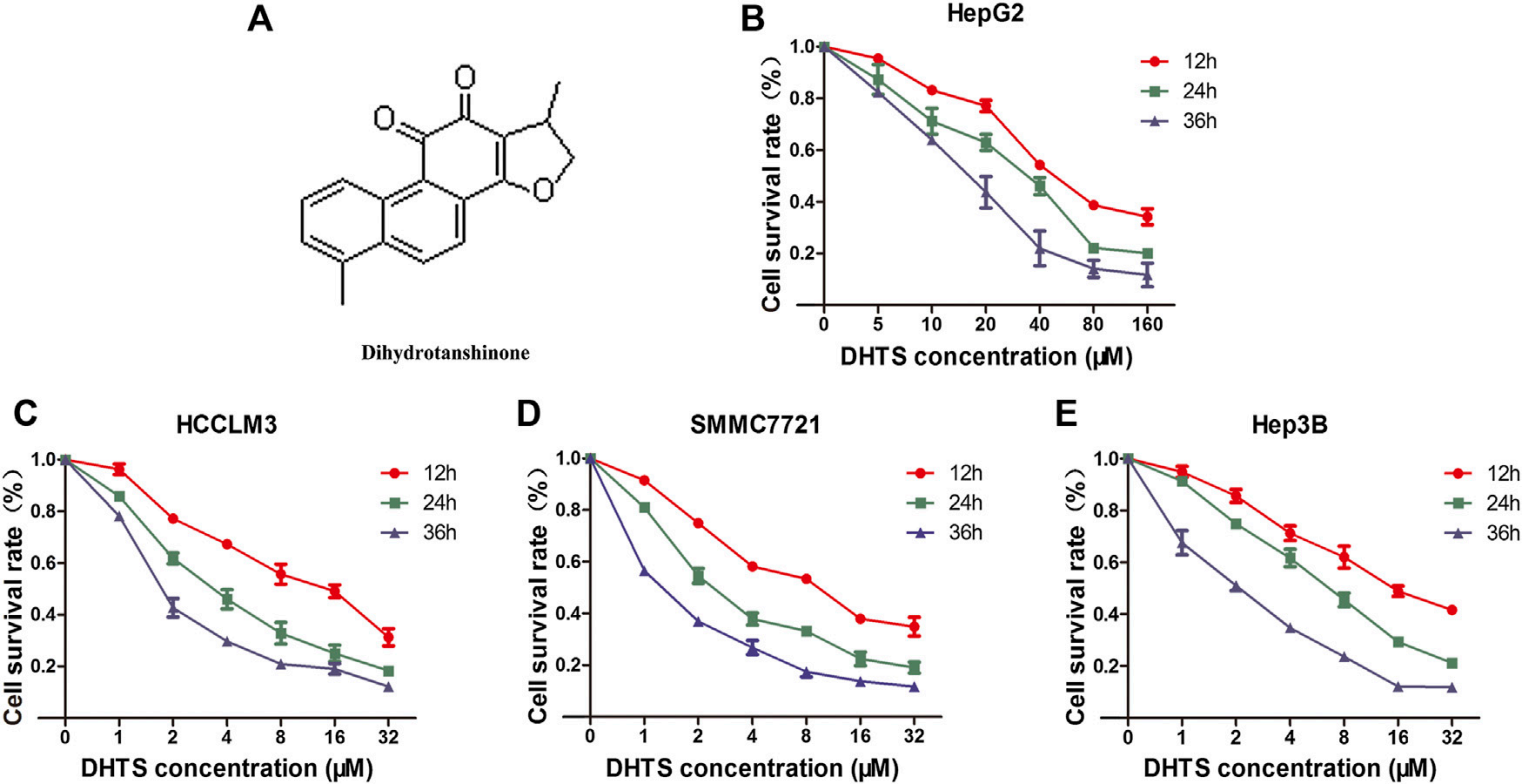
The cell viability of HCC cells was detected by CCK-8 **(A)** Chemical structure of DHTS was used in this study **(B)** Cell viability of HepG2 cells after treatment with DHTS was detected by CCK-8 assay **(C)** Cell viability of HCCLM3 cells after treatment with DHTS was detected by CCK-8 assay **(D)** Cell viability of SMMC7721 cells after treatment with DHTS was detected by CCK-8 assay **(E)** Cell viability of Hep3B cells after treatment with DHTS was detected by CCK-8 assay. Data are presented as means of three experiments, and error bars represent SD, *p < 0.05, compared with the control group.

In order to verify the ability of DHTS in exhibiting inhibitory effects on cell viability. HCC cancer cells HCCLM3, SMMC7721, Hep3B were treated with 1, 2, 4, 8, 16, and 32 μM DHTS. HepG2 cell was cultured with 5, 10, 20, 40, 80,160 μM DHTS. The antitumor effects of the DHTS were detected by CCK-8 assay at different time points (12, 24 and 36 hours). As shown in the cell survival curves, the proliferation of four HCC cell lines were significantly inhibited by DHTS in a concentration- and time-dependent manner (Figures 1B–E). The inhibitory difference of different cell lines maybe the different genetic backgrounds. It is worth noting that the IC50 of SMMC7721 for 24 h is approximately 2 μM, which is lower than the values of the other cell lines. Among the four cell lines, SMMC7721 exerted the best inhibitory effect on the cell viability at 24 h (Figure 1D). Thus, we choose the SMMC7721 by the best inhibitory effect.

### Dihydrotanshinone Induces the Apoptosis of Hepatocellular Carcinoma Cells

**FIGURE 2:**
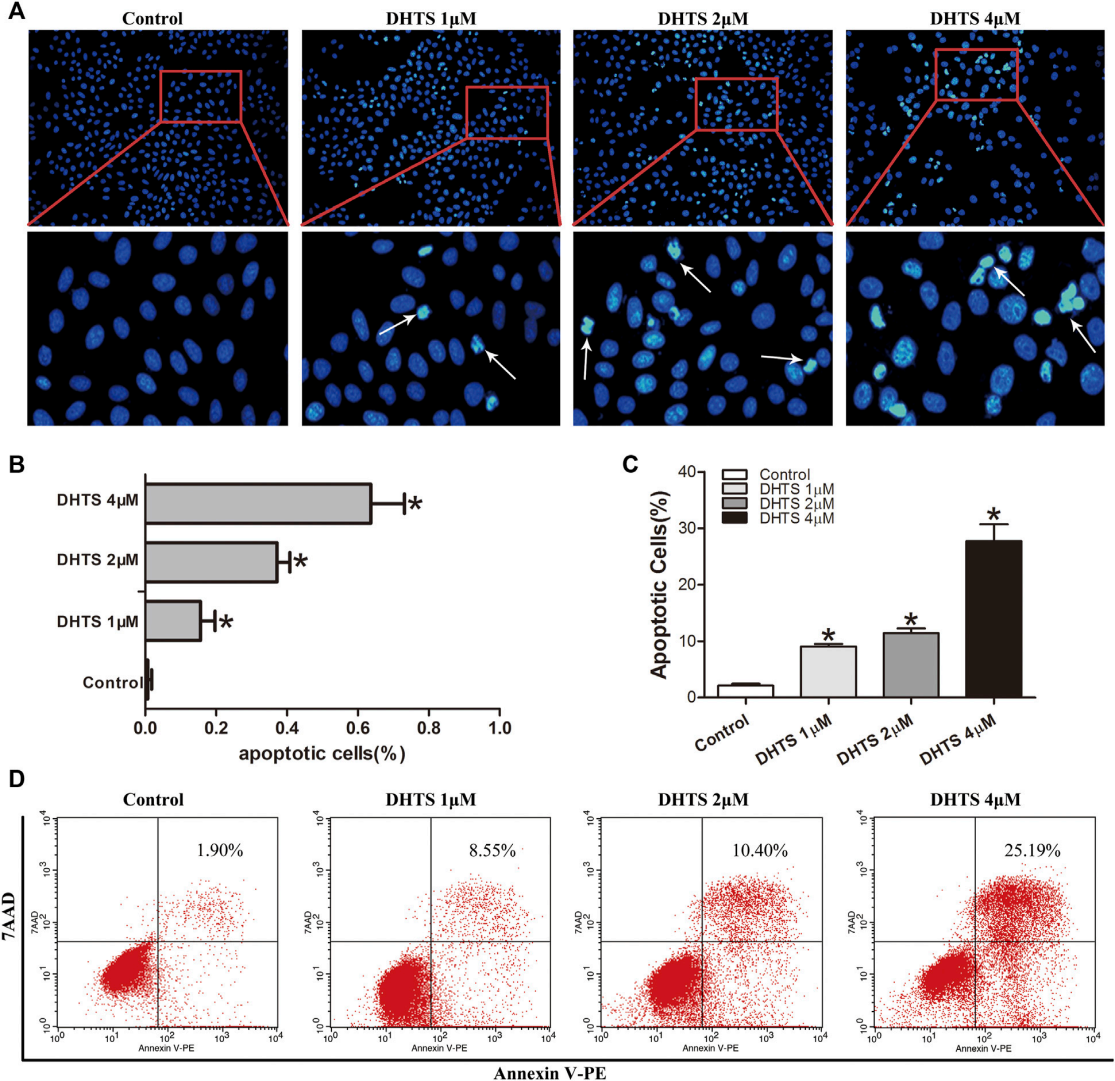
DHTS induces the apoptosis of HCC cells **(A)** Apoptosis was evaluated by Hoechst 33258 straining, and apoptotic features include chromatin condensation, nuclear fragments. Original magnification: ×200 **(B)** Quantification of Hoechst 33258 straining **(C)** Quantification of Annexin V-PE/7-AAD staining **(D)** Apoptosis was detected by Annexin V-PE/7-AAD kit. *p < 0.05, compared with the control group.

Decreased viability of HCC cells after DHTS exposure prompted us to detect the effect of DHTS on apoptosis of cells. As the previous results showed that SMMC7721 cells were more sensitive to DHTS, we selected 24 h as the functional timepoint of apoptosis for SMMC7721 cells treated with DHTS. We performed a Hoechst assay to investigate the morphological characteristics of apoptosis of cells after DHTS exposure. The apoptosis of SMMC7721 cells was induced by the presence of DHTS compared with control (*p* < 0.05) (Figure 2A,B). The apoptotic cells of 4 μM DHTS showed much more condensed nuclei and bright-blue fluorescent than the control medium (*p* < 0.05) (Figure 2A,B). Overall, these results suggest that DHTS may promote the apoptosis of HCC cells.

Staining of SMMC7721 cells with Annexin PE/7-AAD was used to detect the apoptosis of SMMC7721 cells after DHTS exposure 24 h (*p* < 0.05) (Figure 2C,D). The results demonstrated that DHTS could significantly promote apoptosis of cells in a dose-dependent.

**FIGURE 3:**
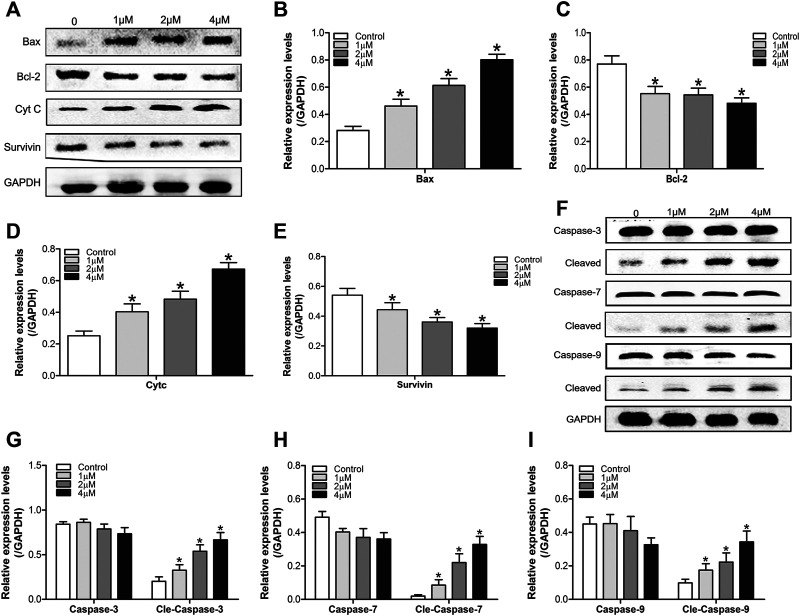
DHTS induces the apoptosis of HCC cells **(A)** Effect of DHTS on the protein levels of Bax, Bcl-2, Cyt C, Survivin **(B)** Quantification of the apoptosis-related protein levels of Bax **(C)** Quantification of the apoptosis-related protein levels of Bcl-2 **(D)** Quantification of the apoptosis-related protein levels of Cyt C **(E)** Quantification of the apoptosis-related protein levels of Survivin **(F)** Effect of DHTS on the protein levels of cleaved caspase 3, cleaved caspase 7, and cleaved caspase 9 **(G)** Quantification of the protein levels of Caspase-3 **(H)** Quantification of the protein levels of Caspase-7 **(I)** Quantification of the protein levels of Caspase-9. GAPDH was used as a loading control. *p < 0.05, compared with the control group.

To further verify the results above, we used western blot to detect the expression of apoptosis-related proteins (Bax, bcl-2, Cyt C, Survivin, and caspase-3,7,9) in SMMC7721 cells treated by DHTS (0, 1, 2, and 4 μM) for 24 h. The results showed that after DHTS treatment for 24 h, the proapoptotic protein Bax gradually increased, and the increase was most significant when DHTS concentration was 4 μM (*p* < 0.05) (Figure 3A,B). At the same time, inhibition of apoptotic protein Bcl-2 was gradually down-regulated with the increase of DHTS concentration (*p* < 0.05) (Figure 3A,C). Cyt C, which can activate caspase-mediated apoptosis, was up-regulated with an increase of dose of DHTS treatment (*p* < 0.05) (Figure 3A,D). The expression of apoptotic inhibitor protein survivin decreased, and the expression level was the lowest when DHTS concentration was 4 μM (*p* < 0.05) (Figure 3A,E). Mitochondrial apoptosis-related protein cleaved caspase-3,7,9 increased significantly with the increase of DHTS concentration (Figures 3F–I). The results indicated that DHTS could promote apoptosis in HCC cell line SMMC7721.

### Dihydrotanshinone Causes Cell Cycle Arrest in Hepatocellular Carcinoma Cells

**FIGURE 4:**
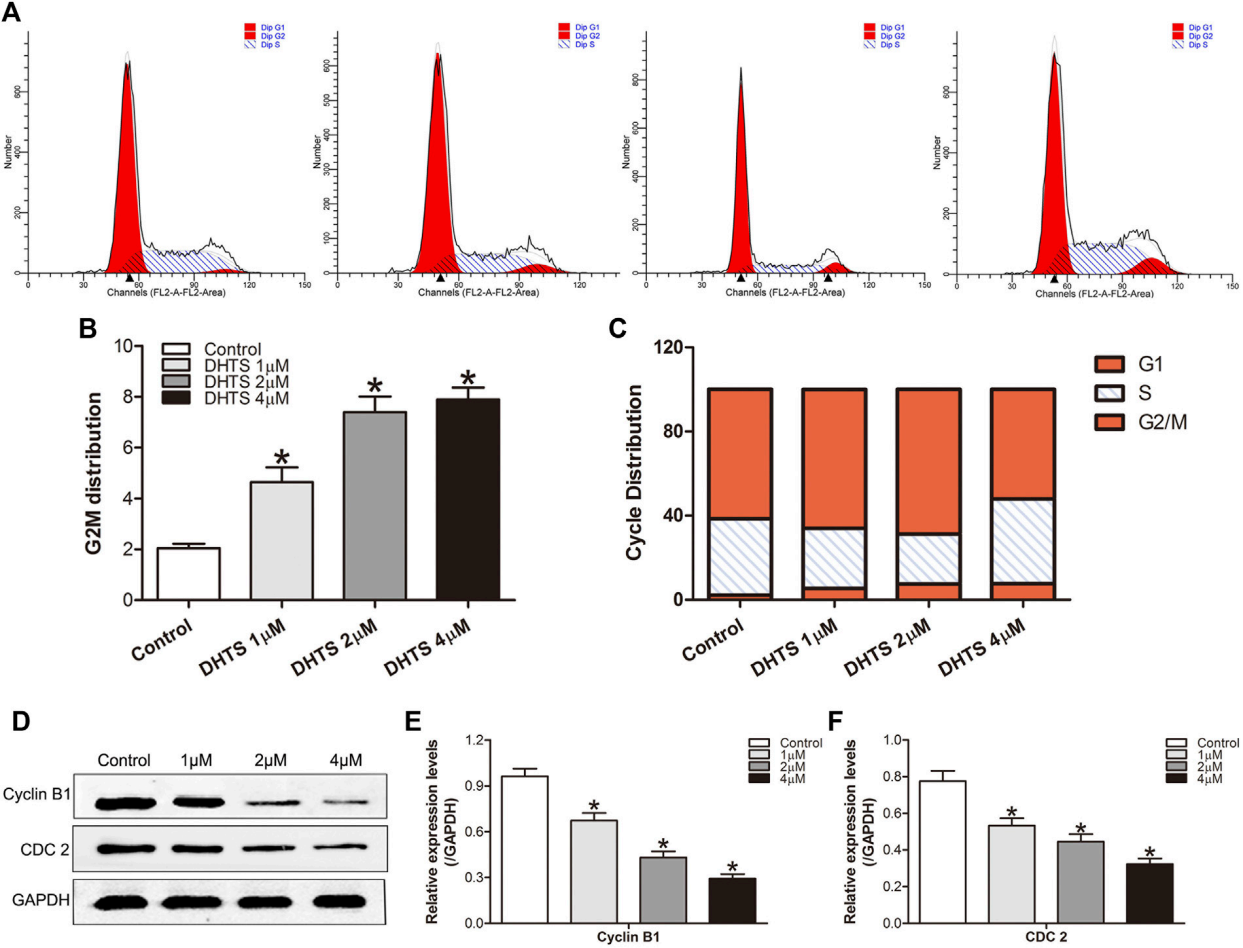
DHTS causes cell cycle arrest in HCC cells **(A)** Induction of cycle arrest in TNBC cells was determined by PI staining **(B)** Representative histograms from flow cytometric analysis in SMMC7721 cells after treatment with DHTS **(C)** The distribution of cell cycle phase in cells after treatment with DHTS **(D)** Effect of DHTS on the protein levels of Cyclin B1, CDC 2 **(E)** Quantification of the apoptosis-related protein levels of Cyclin B1**(F)** Quantification of the apoptosis-related protein levels of CDC 2. GAPDH was used as a loading control. *p < 0.05, compared with the control group.

Next, we measured the effect of DHTS on the growth of SMMC7721 cells. SMMC7721 cells were treated with 0, 1, 2, 4 μM DHTS, respectively. After 24 h, cell cycle phase distribution was evaluated by flow cytometry. The results showed that DHTS induced G2/M cell cycle arrest in SMMC7721 cells (*p* < 0.05) (Figures 4A–C). We observed the accumulation of G2/M phase in SMMC7721 cells when the DHTS concentration was 2 μM. Greatest accumulation in G2/M phase was noted with DHTS 4 μM G2/M DNA damage is an important check point. The checkpoint insures that cells don’t initiate mitosis before they have repaired the damaged DNA after replication. Cells with a defective G2/M checkpoint enter mitosis without repairing their DNA, leading to death after cell division (Lee et al., 2020). DHTS induces G2/M cell cycle arrest in SMMC7721 cells. G2/M is an important DNA damage check-point and numbers of proteins are involved in the regulation of the check-point. Key molecules among them are cyclin B1, CDC 2. We also assessed the levels of these proteins in SMMC7721 cells. Western Blot results showed that DHTS could lead to a decrease in Cyclin B1 and CDC2 levels (*p* < 0.05) (Figures 4D–F). In summary, our data suggests that DHTS-induced reduction in SMMC7721 survival is associated with G2/M-phase arrest and apoptotic cell death.

### Dihydrotanshinone Targets STAT3 and Inhibits JAK2/ STAT3 Activity

**FIGURE 5:**
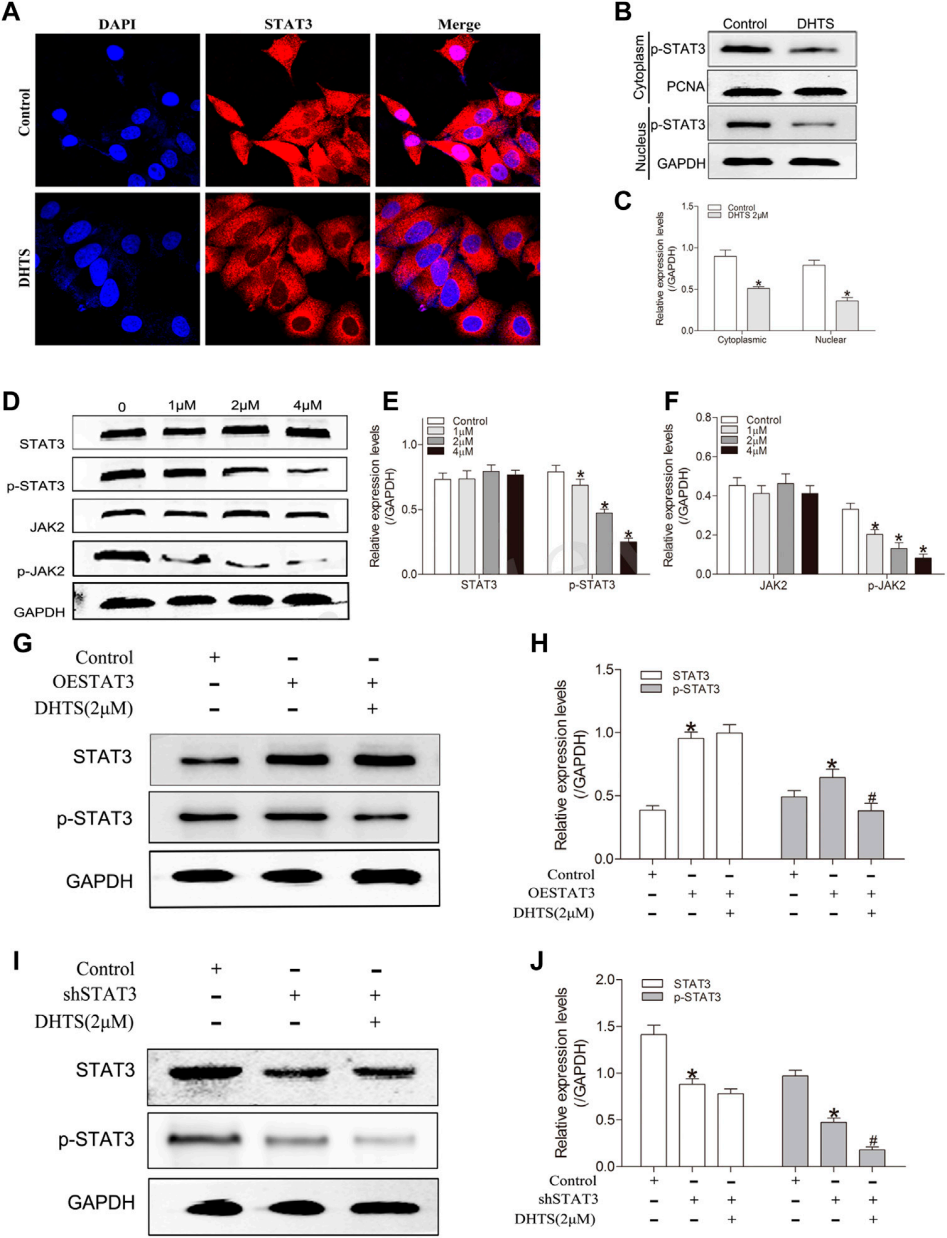
DHTS targets STAT3 and inhibits STAT3 activity **(A)** DHTS inhibited the nuclear translocation of STAT3 in SMMC7721 **(B)** DHTS inhibited the expression of STAT3 in the nucleus of SMMC7721 cells detected by Western blotting **(C)** Quantification of protein levels of p-STAT3 **(D)** Effects of DHTS on JAK2/STAT3 pathway were analyzed by Western blotting **(E)** Quantification of protein levels of STAT3, p-STAT3 **(F)** Quantification of protein levels of JAK2, *p*-JAK2 **(G)** Effects of DHTS on SMMC7721 cells with overexpression of STAT3 were detected by Western blotting **(H)** Quantification of the protein STAT3 and p-STAT3 **(I)** Effects of DHTS on SMMC7721 cells with downregulation of STAT3 were detected by Western blotting **(J)** Quantification of protein STAT3 and p-STAT3. GAPDH was used as a loading control. *p < 0.05, compared with the control group; ^#^ *p* < 0.05, compared with ESTAT3 group in figure3G and shSTAT3 group in figure3I. Abbreviations: ESTAT3, overexpression of STAT3; shSTAT3, downregulation of STAT3.

STAT3 can be involved in the regulation of apoptosis, invasion, migration and angiogenesis. We further performed western blot experiments to verify the effect of DHTS on STAT3. After DHTS treatment, the protein expression of p-STAT3 decreased in a concentration-dependent manner (*p* < 0.05) (Figure 5D,E).

JAK2 is known to stimulate STAT3 phosphorylation on tyrosine Tyr705 in many cancer cells. We speculated that whether DHTS is able to inhibits the phosphorylation of STAT3 by inhibiting the phosphorylation of JAK2. Indeed, DHTS inhibits phosphorylated-JAK2 in a dose-dependent manner (*p* < 0.05) (Figure 5D,F).

We further used immunofluorescence experiment to verify the effect of DHTS on STAT3 nuclear translocation, considering that nuclear translocation is the core of transcription factor function. In the DHTS treatment, STAT3 was inhibited from shifting to the nucleus of the HCC cells (*p* < 0.05) (Figure 5A). In the western blot, SMMC7721 cells were exposed to DHTS for 24 h. Nuclear and Cytoplasmic extracts of DHTS-treated or untreated cells were extracted. After DHTS treatment, the nuclear expression of p-STAT3 decreased (*p* < 0.05) (Figure 5B,C).

The activation of JAK2/STAT3 pathway in response to DHTS was evaluated. The protein expression of phosphorylated-JAK2 and phosphorylated-STAT3, were decreased in DHTS group (Figure 5D). To confirm the effect of DHTS on JAK2/STAT3 signaling in SMMC7721 cells, the STAT3 knockdown and overexpression in SMMC7721 cells were detected. Western-blot showed that protein levels of STAT3 and phosphorylated-STAT3 were higher in STAT3 overexpression SMMC7721 cells (*p* < 0.05) (Figure 5G,H) and lower in STAT3 knockdown SMMC7721 cells (*p* < 0.05) (Figure 5G,H). After DHTS treatment, p-STAT3 was decreased both in STAT3 overexpression and knockdown SMMC7721 cells (*p* < 0.05) (Figures 5G–J).

All results support that DHTS may regulate the proliferation and apoptosis via inhibiting the activation of JAK2/STAT3.

### Dihydrotanshinone Suppressed Tumor Growth of Hepatocellular Carcinoma Cells *in Vivo*

**FIGURE 6:**
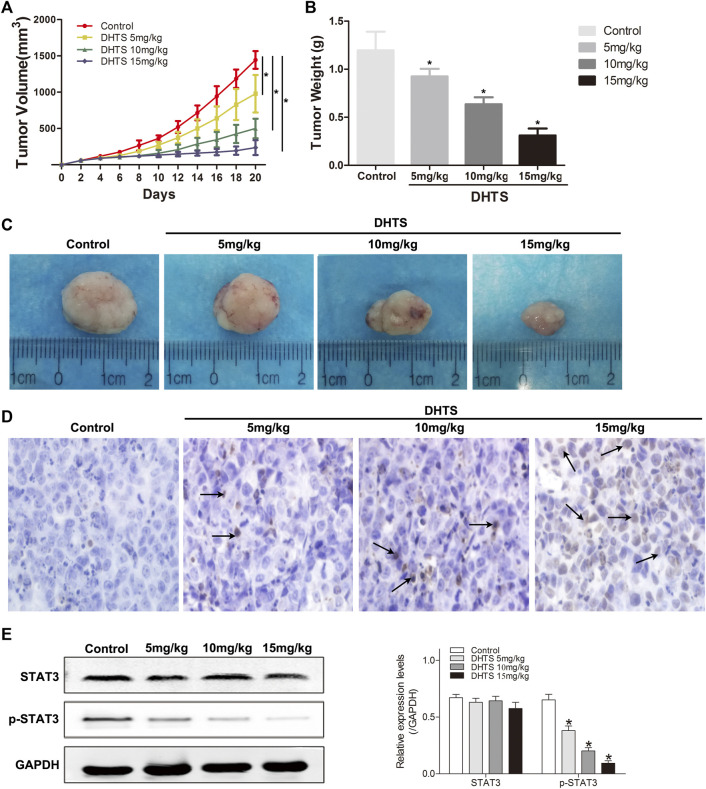
DHTS suppressed tumor growth of HCC cells *in vivo* **(A)** Each time point represents the mean tumor volume for each group **(B)**Tumor weight was obtained at the end of the experiment **(C)** Changes in the tumor volumes of the SMMC7721 xenografts **(D)** Detection of apoptotic cells in tumor tissue by TUNEL assay **(E)** STAT3 and p-STAT3 were detected by Western blotting. Data are presented as means of three experiments, and error bars represent SD, *p < 0.05, compared with the control group.

Our final objective was to determine whether DHTS inhibits the growth of DHTS cells *in vivo*. The SMMC7721 cells were seeded into BALB/C nude mice. Tumor weight and size were significantly reduced in the DHTS groups (at 5, 10 and 15 mg/kg) (*p* < 0.05) (Figures 6A–C). It’s noted that no animal death in all groups. TUNEL staining of tumor tissues of each group showed that efficient apoptosis occurred in the tumor mass from treatment groups, whereas the degree of apoptosis differed in each cluster. Also, 15 mg/kg DHTS group exhibited a distinct apoptosis compared with control (*p* < 0.05) (Figure 6D). Furthermore, we evaluated the level of STAT3 in tumor specimens by Western blot. The results revealed that DHTS decreased the expression levels of p-STAT3 (*p* < 0.05) (Figure 6E). These results indicate that DHTS has significant antitumor effect *in vivo*.

## Discussion

In present study, we evaluated the antitumor effects of DHTS on different HCC tumor cells. DHTS showed excellent antitumor effect in HCC by inhibiting cell proliferation and promoting cell apoptosis. Similarly, DHTS treatment of mice bearing HCC cells showed inhibited tumor growth and increased cell apoptosis. Further experiments showed that DHTS can target STAT3 and play a role in antitumor activity.

In cancer therapy, a principle goal is to kill cancer cells while minimizing death of normal cells. Traditional cytotoxic therapies and the newer agents do this by activating a specific type of programmed cell death–apoptosis (Guo et al., 2019). Both Bax and Bcl-2 belong to the bcl-2 gene family, and Bcl-2 is an apoptotic inhibitor. Bax not only antagonizes the apoptotic effect of Bcl-2, but also has the function of promoting apoptosis. Bax can be transferred from cytoplasm to mitochondrial membrane, at this time, the oligomer of Bax can be combined with Bcl-2 to form a polymer, which can increase the permeability of mitochondrial membrane, thereby increasing the release of CytC, thus starting Caspase cascade reaction and inducing apoptosis (Lin et al., 2011; Campbell et al., 2018; Edlich et al., 2018). Caspases are a family of cysteine-dependent aspartate-directed proteases that play essential roles in regulating cell death and inflammation (Smyth et al., 2020). During this process, orchestrate the demise of cells by the highly distinct regulated cell death phenotypes known as apoptosis and pyroptosis (Ramirez et al., 2018). Caspase-3,7,9 play an essential role in apoptosis (Limpachayaporn et al., 2015; Li et al., 2017). In the present study, the expression levels of Caspase-3, 7,9 in HCC SMMC7721 cells were examined. DHTS effectively increased the expression level of cleaved Caspase-3, 7,9.

We further explored the molecular mechanism of DHTS affecting of apoptosis. Some researchers have proposed that DHTS inhibits breast cancer stem cells through the STAT3 signaling pathway (Kim et al., 2019). Other studies have reported that dihydrotanshinone I alleviates crystalline silicon-induced lung inflammation by regulating Th immune response and inhibiting STAT3 (Zhang et al., 2019). STAT3 is well-known for its critical role in multiple oncogenic processes, such as proliferation, apoptosis, immunity and differentiation (Darnell et al., 1994; Yu et al., 2009). In general, STATs are localized in the cytoplasm in an inactive state. When stimulated by cytokines, STAT3 binds to DNA after entering the nucleus and then participates in the regulation of many essential genes, such as Bax, Survivin, Bcl-2 related to cell survival and proliferation (Ihle et al., 2001). DHTS can target STAT3 and diminish the tyrosine phosphorylation of STAT3 (pSTAT3 at Tyr705) and subsequently affects the STAT3 dimerization and entry into the nucleus. In our research, we found that DHTS inhibits STAT3 and translocation from the cytosol to the nucleus.

DHTS is a component of the well-known traditional Chinese medicinal plant *Salvia miltiorrhiza* and is used to treat cardiovascular disease, inflammation, hepatitis, and cancer (Yu et al., 2004; Wang et al., 2007). Studies showed that the toxicity of Chinese *Salvia* plant use for herbal prescriptions is considered safe (Xu et al., 2018). Previous studies have also shown that DHTS can leads to growth inhibition and apoptosis in tumor cell lines as well as in xenograft models (Kim et al., 2019; Tan et al., 2019). *In vivo* studies of DHTS, Wang et al. found that DHTS reversed metabolic reprogramming in colon cancer by regulating PTEN/Akt/HF1α mediated signaling pathways (Wang et al., 2018). Liu et al. proposed that DHTS showed anticancer activity in human HL-60 leukemia cells *in vivo* (Liu et al., 2015). Cai et al. showed that DHTS inhibit hemangiomas *in vitro* and *in vivo* by inducing pro-apoptotic and anti-angiogenic mechanisms (Cai et al., 2018). We also investigated the effect of DHTS on HCC *in vivo*, the results were consistent with the above reports, and DHTS inhibited the proliferation of HCC cells and promoted their apoptosis. Some previous *in vivo* studies have reported only one or two concentrations of DHTS. However, in our study, three groups of DHTS with different doses were treated separately, and with the increase of DHTS concentration, the volume and weight of the transplanted tumor gradually decreased. Moreover, the therapeutic effect of DHTS was confirmed by the TUNEL staining of tumor sections, which presented remarkable cell apoptosis in tumor mass. Other studies have reported that a lot of malignant transformations were associated with constitutive STAT3 activation (Swiatek-Machado et al., 2020; Yu et al., 2004). Similarly, we found that DHTS inhibited HCC cells growth, increased HCC cells apoptosis, and decreased phosphorylated STAT3 levels *in vitro* and *in vivo*. In our vivo studies treatment with DHTS dramatically reduced the levels of phosphorylated STAT3.

In conclusion, our study suggests that DHTS can effectively inhibit cell proliferation and increase cell apoptosis via blocking the activation of JAK2/STAT3 pathway. This study revealed that the potential clinical role of DHTS in the treatment of HCC.

## Data Availability

The raw data supporting the conclusions of this article will be made available by the authors, without undue reservation.
